# How an Outbreak of COVID-19 Circulated Widely in Nepal: A Chronological Analysis of the National Response to an Unprecedented Pandemic

**DOI:** 10.3390/life12071087

**Published:** 2022-07-20

**Authors:** Basu Dev Pandey, Mya Myat Ngwe Tun, Kishor Pandey, Shyam Prakash Dumre, Khin Mya Nwe, Yogendra Shah, Richard Culleton, Yuki Takamatsu, Anthony Costello, Kouichi Morita

**Affiliations:** 1 Department of Molecular Epidemiology, Institute of Tropical Medicine, Nagasaki University, Nagasaki 852-8523, Japan; 2 Department of Virology, Institute of Tropical Medicine, Nagasaki University, Nagasaki 852-8523, Japan; myamyat@tm.nagasaki-u.ac.jp (M.M.N.T.); bb55418851@ms.nagasaki-u.ac.jp (K.M.N.); yukiti@nagasaki-u.ac.jp (Y.T.); moritak@nagasaki-u.ac.jp (K.M.); 3 Central Department of Zoology, Tribhuvan University, Kathmandu 44618, Nepal; kishor.pandey@cdz.tu.edu.np; 4 Central Department of Microbiology, Tribhuvan University, Kathmandu 44618, Nepal; shyam.dumre@cdmi.tu.edu.np; 5 Seti Provincial Hospital, Kailali 10900, Nepal; yshah@kist.edu.np; 6 Division of Molecular Parasitology, Proteo-Science Center, Ehime University, Matsuyama 790-8577, Japan; culleton.richard.oe@ehime-u.ac.jp; 7 Institute for Global Health, University College London, London WC1N 1EH, UK; anthony.costello@ucl.ac.uk

**Keywords:** COVID-19, Nepal, pandemic, vaccine

## Abstract

Coronavirus disease 2019 (COVID-19) is caused by the severe acute respiratory syndrome coronavirus 2 (SARS-CoV-2). The first COVID-19 case was reported in Wuhan, China, in December 2019. In March 2020, the World Health Organization (WHO) declared COVID-19 a global pandemic. The first COVID-19 case in Nepal was reported in January 2020 in a Nepalese man who had returned from Wuhan to Nepal. This study aims to evaluate the government of Nepal’s (GoN) response to the COVID-19 pandemic and explore ways to prevent COVID-19 and other pandemic diseases in the future. As of May 2022, a total of 979,140 cases and 11,951 deaths associated with COVID-19 have been reported in Nepal. To prevent the spread of the virus, the GoN initiated various preventive and control measures, including lockdown strategies. The effects of COVID-19 are expected to persist for many years; the best strategies a resource-limited country such as Nepal can implement to control pandemic diseases such as COVID-19 in the pre-vaccine stage are to increase testing, tracing, and isolation capacity.

## 1. Introduction

COVID-19, caused by the severe acute respiratory syndrome coronavirus 2 (SARS-CoV-2), was first reported in Wuhan, China, in December 2019 and is now a global pandemic [1]. Despite substantial efforts to manage and control the pandemic, morbidity, and mortality due to COVID-19 and its variants have led to significant alterations in daily life [2]. Following its identification, SARS-CoV-2 continued to evolve different variants, including the most recent variant of concern, Omicron (B.1.1.529), identified during a sudden surge of cases [3,4]. The World Health Organization (WHO) classified Omicron as a variant of concern on 26 November 2021, adding it to the earlier variants Alpha, Beta, Gamma, and Delta [4,5]. Continuous viral evolution, the lack or limited availability of vaccines, and wave after wave of outbreaks have posed significant challenges to resource-limited countries such as Nepal in responding to the pandemic [6,7].

A Nepalese man, who had returned from Wuhan, China on 13 January 2020, visited the Sukraraj Tropical and Infectious Disease Hospital (STIDH), Kathmandu, complaining of a cough. He was admitted to the hospital, and efforts were made to exclude common infectious diseases prevalent in Nepal. Real-time reverse transcription polymerase chain reaction (RT-PCR) assays for influenza A and B viruses and NS1 antigen rapid tests for dengue viruses, scrub typhus, malaria, and *Brucella* were negative. There was no laboratory facility for SARS-CoV-2 testing in Nepal at the time, so the samples were sent to the WHO laboratory in Hong Kong, where they were confirmed positive for SARS-CoV-2. Full genome analysis showed that the samples were closely related to the strain from Wuhan [4,8]. On 23 January 2020, the Ministry of Health and Population (MoHP) officially declared this as the first case of COVID-19 in Nepal. The second case was detected on 17 March 2020. Shortly afterward, the virus spread rapidly throughout the country [9].

The COVID-19 pandemic remains a devastating crisis for Nepal, partially due to the fact that the country was poorly prepared for this unprecedented situation [10]. This was evident from the awareness and preparedness levels of Nepalese and international health care professionals at the onset of the pandemic [11]. Nepal had never experienced such an event in the past and its limited capacity to respond was detrimental to containment efforts [12]. The country was severely affected, with an ongoing and steady increase in morbidity and mortality of COVID-19 cases more than two years after the first case was diagnosed. As of May 2022, 5,703,008 PCR assays had been performed throughout the country, detecting a total of 979,140 COVID-19 cases. There have been 11,952 deaths until May 2022. The recovery rate was 98.8%, with a case fatality rate of 1.2%. To comprehend how SARS-CoV-2 spread so widely in Nepal and to prepare for future health crises, a clear and accurate understanding of the series of key early epidemiological events including analysis of the concerned authorities’ responses to the emergence of COVID-19 must be established. Towards this aim, we present a chronology of key events and actions pertaining to the pandemic from 23 January 2020, when the first case was identified in Nepal, until May 2022, when cases were appearing all over the country has been analyzed.

Based on these findings, we suggest that improving the speed of disease early detection and the implementation of alerts will help the country respond more efficiently to future emergencies of national and global health concern. Establishing the chronology of major events also permits an assessment of the national effort to detect and prevent transboundary diseases and threats to health under the obligations of the WHO’s International Health Regulations. Using a conceptual framework for key stages from outbreak to pandemic and lockdown after the second case was confirmed in March 2020, we delineated the series of identified actions to establish how the health systems functioned and pinpoint potential areas and gaps for improvement in the early outbreak alert and response systems [13,14]. Based on these analyses, we propose a set of options for preparing resilient health systems to respond to high-impact pathogens of international concerns with specific features such as those of SARS-CoV-2. These proposals will ultimately support the design and implementation of pandemic preparedness and response frameworks.

## 2. Materials and Methods

This is a descriptive epidemiological study of COVID-19 in Nepal. Nepal is a landlocked country bordered by India in the east, west, and south and by China in the north and is home to approximately 30 million people. Geographically, Nepal is divided into three regions, the Terai, hills, and mountains. Politically, it is divided into seven provinces, 77 districts, and 753 municipalities.

**Figure 1:**
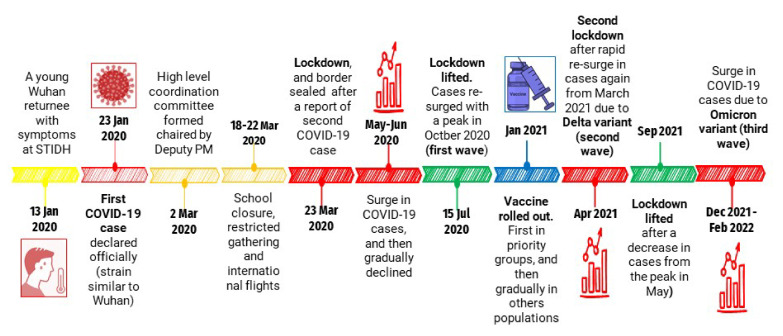
Chronology of key events of COVID-19 in Nepal.

The COVID-19 data for this study were obtained from official situation reports of the MoHP of the government of Nepal (GoN), which are publicly available. Data were collected from 23 January 2020 to 31 May 2022 (Figure 1). Data on daily COVID-19 cases are available from the MoHP’s website. Data were analyzed in Excel 2019 (Microsoft, Los Angles, CA, USA).

## 3. Results

### 3.1. Early Detection of COVID-19 Cases in Nepal

The first SARS-CoV-2 infected case in Nepal was reported on 23 January 2020 in a Nepalese man who had returned to Kathmandu from Wuhan, China (Figure 1). He had symptoms on 3 January 2020, six days before flying back to Nepal. He presented at the outpatient department of Sukraraj Tropical and Infectious Disease Hospital (STIDH), Kathmandu, on 13 January 2020 with a cough; physicians suspected the possibility that the patient was infected with a novel virus that was at the time causing an outbreak of pneumonia in Hunan, China. The patient was immediately admitted to hospital and tested for panel of common infectious diseases using RT-PCR assays for influenza A and B viruses and NS1 antigen rapid tests for dengue viruses, and serological tests for scrub typhus, malaria, and *Brucella*, but all tests were negative. The hospital director called an emergency meeting with the director of the Epidemiology and Disease Control Division (EDCD), the director of the National Public Health Laboratory (NPHL), a representative from the WHO, and the treating physician. As per the meeting decision, throat swabs were collected and sent to the WHO laboratory in Hong Kong for real-time RT-PCR assays for COVID-19 and were found to be positive. On 23 January 2020, the MoHP officially declared the patient Nepal’s first COVID-19 case [3]. All 15 staff members working in the special isolation ward of STIDH who had been in close contact with the patient were kept under observation for 14 days. However, none of them showed clinical symptoms during that period. The NPHL’s capacity for COVID-19 testing was enhanced by the implementation of using RT-PCR technology for the first time in Nepal.

### 3.2. The Government’s Initial Response to COVID-19

Prompt action was taken by the MoHP to strengthen the country’s response to prevent the spread of the virus transmission, mainly through the strengthening of health desks at Tribhuvan International Airport and then at other domestic airports. The ground points of entry on the Nepal–China and the Nepal–India borders were also equipped and strengthened with additional health desks. Travel restrictions were imposed on both sides of these borders [4]. In addition to these initial measures, the GoN sought to repatriate Nepalese nationals from Wuhan, China, resulting in the return of 175 Nepalese citizens. They were quarantined for 14 days and permitted to return to their homes following negative tests. The MoHP activated the Incident Command System (ICS) and Health Emergency Operation Center (HEOC) and initiated coordinated efforts to respond to the virus. The High-Level Coordination Committee (HLCC), led by the Deputy Prime Minister, was formed on 2 March 2020, and subsequently made several crucial decisions intended to limit the spread of COVID-19.

### 3.3. Detection of a Second Case and Announcement of Lockdown

No cases were reported until 17 March 2020, when a Nepalese person who had returned from France was found to be carrying the virus [4]. There was widespread public concern about the country’s diagnostic capabilities as no cases were detected from 23 January 2020 to 17 March 2020. On 18 March 2020, schools were closed, and gatherings of more than 25 people were discouraged. On 22 March 2020, the GoN prohibited all international flights and suspended operations of nonessential businesses and domestic long-distance transportation. All international borders were closed on 23 March 2020. The GoN issued a nationwide lockdown from 24 March 2020 to 21 July 2020, imposing a ban on domestic and international travel and closing borders and nonessential services. There were only two COVID-19 cases and no fatalities recorded at the beginning of the nationwide lockdown.

### 3.4. Strengthening the System

During the initial phase of the outbreak, PCR tests for SARS-CoV-2 were limited to only at the NPHL, with samples sent from all over the country to Kathmandu. The laboratories were gradually expanded to include the BP Koirala Institute of Health Sciences and public and private laboratories in all seven provinces. Sudarpaschim province borders India, and the provincial government there established seven molecular laboratories. Within a year, starting with one public-sector COVID-19 diagnostic laboratory at the NPHL, the MoHP enhanced a network of more than 100 COVID-19 testing facilities across the country in both the public and private sectors [4]. Antibody-based rapid diagnostic tests (RDTs) were used for screening purposes, particularly at points of entry. However, false-positive and -negative reporting led to discrepancies and the withdrawal of this RDT by the MoHP. On 29 March 2020, the COVID-19 Crisis Management Centre (CCMC), was formed and chaired by the Deputy Prime Minister to serve as the implementing authority of the HLCC.

### 3.5. Information Management and Risk Communication

After issuing strict lockdown orders, the GoN initiated an effective flow of information to the general public in order to enhance risk awareness and community participation. The MoHP started publishing daily live press briefings in the third week of March. A spokesperson provided daily updates on the situation to ensure a uniform flow of information from the press to the public. The MoHP communicated through various routes, including mobile applications, call centers, hotlines, and social media (Facebook and Viber; MoHP; https://covid19.mohp.gov.np/). The EDCD developed and implemented telephone-based hotlines that received several thousands of calls a day from the public, and these were soon expanded and made available 24 h a day. Educational materials were developed and distributed widely to disseminate messages. On 9 April 2020, the Health Cluster for COVID-19 was activated, headed by the ICS coordinator and co-led by the WHO. With this activation, regular cluster meetings were held every Thursday with health officials at the federal and provincial levels and representatives from private partnerships. Subsequently, health cluster and provincial meetings were held separately to make the discussions more efficient.

### 3.6. Case Management

The government designated STIDH as the primary hospital for COVID-19 case management, with the Patan Academy of Health Sciences and the Armed Police Forces Hospital as secondary hospitals. Clinical guidelines for case management and standard operating procedures were prepared and distributed by the EDCD. Later, as COVID-19 cases continued to surge, the MoHP designated the Army hospital, the facilities of the Ayurvedic Hospital Research and Training Center, and Nepal Medical College as COVID-19 treatment facilities and converted Bir Hospital into a COVID-19 special unified hospital. Specific locations were identified to be use as quarantine centers across the country; however, the number of centers was insufficient, and several were mismanaged. As the provision of health services and clinical management are key to saving lives, the MoHP held a series of consultations with the central hub hospitals to prepare for appropriate case management. As the 25 country-wide central hospitals were unable to respond to an international public health emergency such as the COVID-19, the MoHP designated over 150 more hospitals, including private hospitals, as COVID-19 hospitals upon the recommendations of the central and cluster meetings. Hospital services were provided free of charge, and funds were provided to the hospitals according to the number and severity of the cases they managed. Initially, all arrangements for patient admission, including contacting the infected person, arranging an ambulance, finding a bed, and transport to the hospital, were made by an EDCD case-management team. The MoHP, the Curative Services Division led a multi-sectoral and multi-partner team to design and develop a rapid assessment tool to evaluate 12 selected COVID-19 level II hospitals in April 2020. Gaps were identified in multiple areas, including intensive care unit (ICU) capacity, the number of ventilators, infection prevention and control (IPC) measures, logistics, human resources, and training. Training for all health care professionals and non-technical staff, a dedicated IPC budget, and ongoing non-COVID-19 services were recommended.

### 3.7. Data Management

Initially, when only a few cases were being reported, the NPHL sent information directly to the EDCD, where the data were maintained in Excel. Data received at the EDCD were processed and sent to the MoHP for daily press briefings to the media and for publication. However, as cases began to surge rapidly and several laboratories started COVID-19 testing and reporting data handling became challenging. Therefore, other resources such as Go Data, developed by the Global Outbreak Response Network (GOARN) of the WHO, were utilized. Multiple centers maintained information, including the CCMC and the Home Ministry; these multiple sources led to conflicting information that created discrepancies in data.

In April 2020, COVID-19 hospitals, testing facilities, Health Directorates, Provincial Health Emergency Operations Centers (PHEOC), and the Ministry of Provincial Social Development Ministry all started to observe a surge in cases. This necessitated the development of a uniform reporting system and a template was immediately shared among the stakeholders. However, challenges emerged due to low compliance with the instructions regarding the use of the reporting template and stakeholders’ data sharing with the EDCD in multiple formats, such as scanned copies, Excel sheets, or direct emails. Further confusion in data sharing was created because the PHEOCs, the hospitals, and local health facilities reported data to both the EDCD and the HEOC, which led to challenges in verifying the information. In some cases, the provincial and local health agencies hesitated to share data because they believed they did not come under the jurisdiction of the MoHP. The health management information system established an Information Management Unit to ensure the systematic flow of data. The Nepali Army was responsible for managing the deceased and maintaining data pertaining to them, which it provided to the MoHP and the EDCD. However, the data regarding the deceased reported by health facilities to the EDCD differed from the Nepali Army’s reports as the definition of death from COVID-19 differed between these institutions.

### 3.8. Case Investigation and Contact Tracing

**Figure 2:**
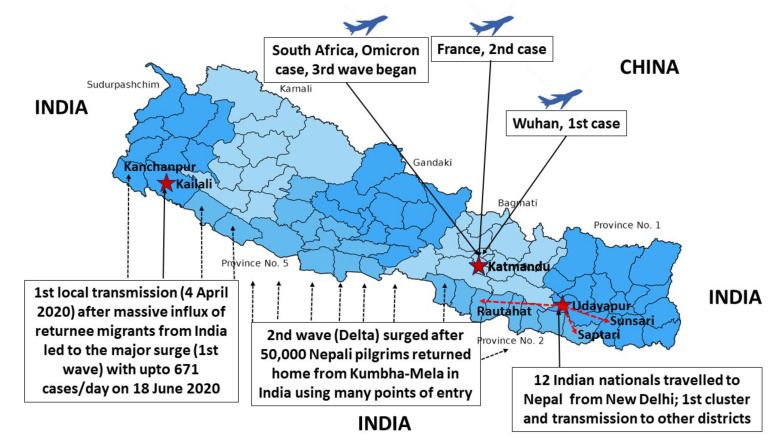
Map showing various surges of corona entering in Nepal.

On 4 April 2020, the first case of local transmission was confirmed in a 34-year-old women from Kailali District (Figure 2) [4]. A group of people had traveled to New Delhi, India, where they attended a conference, on March the 8th and 9th, and then returned to Nepal via Birgunj, on 11 March 2020. On 17 April, 12 Indian nationals from New Delhi quarantined in a mosque in Bhulke, Udayapur district, tested positive for COVID-19. These people traveled to other parts of the eastern Terai, including Sunsari, Saptari, Rautahat, Parsa, and Udayapur districts, from 30 March to 13 April 2020. The first death due to COVID-19 in Nepal was that of a 29-year-old pregnant woman on 14 May 2020; and by November 2021, 11,496 deaths had been reported [15].

Initially, the EDCD formed case investigation and contact tracing (CICT) teams, consisting of doctors, public health officers, and laboratory personnel, and these teams were mobilized for each case. However, as case numbers started to increase, the EDCD coordinated with the district health office team and local authorities in Kathmandu Valley for CICT. In August, meetings were held with the HLCC and mayoral forums for the prevention and control of COVID-19. To systematize CICT, measures were initiated with microplanning in meetings with mayors, deputy mayors, district COVID-19 focal persons, municipal health officials, and EDCD officials. These meetings resulted in the EDCD designating health coordinators for municipalities. As the number of cases increased, it became impossible to conduct contact tracing from the central level to the local level. In May 2020, the GoN formulated the Preparedness and Response Plan to prevent and minimize the spread of COVID-19. The plan, which was endorsed by the cabinet, also suggested forming 1075 CICT teams at the local level that would constitute members from the public health, laboratory, nursing, local municipality, administrative staff, and security personnel [16]. The local government was primarily responsible to establish and manage the quarantine centers in each municipality with the support from the government and partial involvement of private organizations as per the guidelines. When the pandemic began to surge in Nepal during March to July 2020, there were 8241 quarantine centers and 238 holding centers across the country [17]. In the beginning, most of the quarantine centers were located at the points of entry along the Indo-Nepal border using school buildings, government facilities and hotels. These centers were converted to the holding centers for the returnees as well as the isolation centers for the symptomatic cases later [18].

### 3.9. The Initial Surge of the First Wave of COVID-19

**Figure 3:**
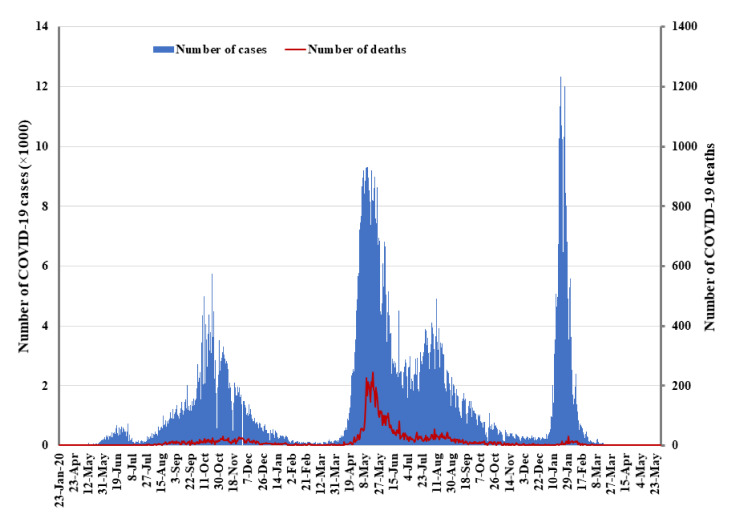
Number of COVID-19 cases and deaths in Nepal.

The pandemic may have blurred international boundaries and brought much of the world closer together, but less so for Nepal, one of India’s neighbors. In fact, Nepal’s decision to publish new maps that included areas of dispute with India cooled relations between the countries [19]. The publication by the Minister for Land Management, Cooperatives and Poverty Alleviation of Nepal on 20 May 2020 sparked a dispute between the countries. The dispute was further heated by a high-level parliamentarian’s speech that stated that the Indian virus seemed more lethal than the Chinese and Italian viruses. At the same time, toward the end of March, India implemented one of its strictest lockdowns. Thousands of Nepalis working across India, mostly young men, had traveled long distances in the hope of returning to their homes in Nepal [16]. They had been waiting for weeks at the Indo–Nepal border due to travel restrictions imposed by the GoN. The decision to let them enter the country came too late, probably causing a surge of COVID-19 cases in the Sudurpaschim and Madhesh provinces of Nepal. Despite continuous efforts, Nepalese authorities at the central, provincial, and local government have been unable to systematically and efficiently quarantine returnees and migrants from other countries, especially from India. The inflow of COVID-19 cases through the multiple points of entry across Indo–Nepal borders resulted in the first surge, leading to as many as 671 cases in a day on 18 June 2020 (Figure 3). In June and July 2020, there were over 200 daily cases on average, but there were few deaths [20].

The GoN made a decision to lift the four-month-long lockdown on 22 July 2020, with a few restrictions in place. Initially, all domestic and international flights were permitted on 17 August 2020; however, it then extended restrictions to 31 August 2020. In July and August 2020, Nepal encountered an unexpected rapid rise in cases, and deaths increased every day. In the first wave, the average number of daily cases rose to 3000, and deaths peaked at fewer than 20 a day. Cases peaked again in October 2020, with 30 deaths on 4 November 2020. The first wave peaked in late October at 5743 cases per day, with a case death rate of less than 1% (13). The number of cases gradually decreased and dropped to a minimum level in January 2021. Although cases reached a baseline level in February of 2021, deaths from earlier infections may have led to a rise in the death rate.

### 3.10. The Second Wave of COVID-19 in Nepal and the Response

In March 2021, the new Delta variant, labeled as more virulent and infectious by the WHO, was first reported in India [19]. Throughout the spring, hundreds of thousands of people gathered in the streets, engaging in political campaigns to prepare for the May 2021 election and adding to the number of people already congregating for seasonal weddings and festivals. In mid-April, when cases in India were constantly rising, an estimated 50,000 Nepali pilgrims travelled to northern India for Kumbh Mela, a Hindu festive gathering that draws millions of people [19]. While there, many of the pilgrims caught COVID-19, perhaps triggering the second wave in Nepal. The second wave began in April 2021, and cases peaked in May, when the average number of cases per day reached 9317 (on 11 May 2021), and deaths peaked at approximately 246 a day. These numbers coincided with those in India, where the Delta variant contributed to a surge with 414,188 cases recorded on 6 May 2021, setting a global record [19]. On 29 April 2021, the Kathmandu Valley and other parts of Nepal returned to lockdown until 1 September 2021. The existing government facilities were not able to handle the volume of sick patients, and hospitals continued to experience widespread shortages of oxygen and other essential logistic supplies, leading to potentially avoidable losses of life [21]. Cases gradually decreased after June, with an infection rate of 2% in the confirmed cases per day at the end of November 2021. According to the second nationwide seroprevalence survey, between 5 July 2021 to 14 August 2021, over two-thirds of the Nepali population carried antibodies against COVID-19, up from 14.9% in the first seroprevalence survey conducted by the EDCD from 9 October to 20 October 2020 [22,23].

### 3.11. The Introduction of the Omicron Variant in Nepal

The number of new cases per day began to decline in December 2021 from the peak of the second wave on 11 May 2021. On 6 December 2021, the Omicron (B.1.1.529) variant was observed for the first time in Nepal, merely two weeks after it was first identified in South Africa on 24 November 2021. The appearance of Omicron led to a surge in daily new cases that peaked on 20 January 2022, reaching a record of more than 10,000 cases [17]. The third wave ended after two months (by February 2022). The number of deaths per day (*n* = 32) was much lower in the third wave compared to those of the second wave (*n* = 246) [17]. As during previous waves, the long (1800 km), open border between India and Nepal featured inadequate monitoring of compliance with public health protocols, and may have contributed to the size of the third wave. The lessons learned from the first and second waves combined with the introduction of COVID-19 vaccination may have contributed to the management of the third wave in Nepal. Currently, there are few daily new cases and zero deaths reported in the country. Apart from school closures, limitations on mobility, and business operation strategies, the GoN greatly facilitated COVID-19 vaccinations among the population, including in children aged 12–17 years old. S gene target failure or “S gene dropout”, producing a false-negative result, has been used as a proxy marker to screen for Omicron, and this option is available in several COVID-19 laboratories throughout Nepal.

### 3.12. COVID-19 Vaccination

In Nepal, COVID-19 vaccination started on 27 January 2021, with the COVISHIELD vaccine imported from India. As of 18 January 2022, only 40% of Nepal’s 30 million people had been fully vaccinated, while approximately 53% had received at least one dose. However, vaccination coverage was quickly extended over the proceeding months. As of 19 April 2022, 75.9% of the population had received at least one dose of the vaccine, while 66.4% had received two doses. For those within six months of their second dose, 2,474,879 doses of additional vaccine (booster shots) had been administered at the time of writing [24]. No vaccinations have been initiated for children below 12 years of age. Nepal used six different vaccine formulations: Vero Cell, COVISHIELD, AstraZeneca (Japanese and Swedish), Moderna, Pfizer, and Janssen [25].

## 4. Discussion

This analysis of Nepal’s early response to the COVID-19 pandemic from 23 January 2020 to May 2022 highlights crucial lessons and clarifies potential pathways for improving future pandemic preparedness and response. Nepal neighbors China, where the novel coronavirus was first detected on 31 December 2019 [26]. Within a month, on 23 January 2020, Nepal had detected its first case in a young Nepali citizen who had returned from Wuhan, China [3]. Nepal responded quickly and imposed a lockdown after detecting a second COVID-19 case on 24 March 2020; the lockdown lasted almost six months, paralyzing the country [27].

Nepal initiated an early lockdown in order to buy time to prepare the country to respond to the pandemic [28]. This approach to lockdown differed to that applied in other countries, in which restrictions on the population were imposed only after significant numbers of deaths had occurred [29]. Although there is, perhaps, an argument to be made for a universal, WHO-sanctioned protocol for the implementation of lockdown measures at a country level during a pandemic, differences between countries at the political, population and environmental levels make this unrealistic.

The vast majority of early cases in Nepal were in young men who had returned from India; they were usually asymptomatic, and the case fatality rate was initially low at 0.5%, increasing to 1.4% in the second wave [15]. Cases were detected in Kathmandu and a few neighboring districts until March 2020, but quickly thereafter, spread over 77 districts, and a seroprevalence study showed a 14.9% prevalence of antibodies against SARS-CoV-2 by September 2020 [22].

Even in the urban areas such as Kathmandu, over one-quarter of people did not use masks while another 25% did not use them properly during the pandemic. Social distancing was not followed in the public places including in the hospitals, public vehicles and offices [18]. On the other hand, even though there is very high level of knowledge on preventive measures, relatively less practice and no or minimal compliance were observed in areas bordering India [30]. Efforts on enhancing practices of public health measures using face masks, hand washing and maintaining physical distance by the government are key measures to contain the unprecedented transmission.

The first case was confirmed by a laboratory in Hong Kong, and Nepal was unprepared to diagnose a novel disease due to its lack of molecular laboratories. Later, the NPHL installed RT-PCR facilities; more than 100 are now active, but there was a severe shortage of laboratory reagents, logistics, and manpower to operate them during the first wave [31]. At the same time, a lack of face masks and sanitizer created difficulties in implementing public health measures [32]. Unavailability of the face masks, sanitizers, and personal protective equipment (PPEs) was a major issue in the initial stage of the pandemic. However, at least one or more Water Sanitation and Hygiene (WASH) services including hygiene kits, facemask, soap, and sanitizers were provided to 199,252 people and 148,720 returnees in collaboration with partner organizations in the first and second waves of COVID-19 in Nepal [18].

There were few isolation facilities for case management, and STIDH, designated as the primary hospital for COVID-19 management in the beginning, had only five beds with isolation capacity. The health sector emergency response and preparedness plan showed 1595 ICU beds and 840 ventilators available in 194 hospitals [1]. Later, the MoHP designated 111 hospitals to operate COVID-19 clinics and 28 hospitals to treat COVID-19 cases, and Bir Hospital was designated as the COVID-19 Unified Hospital. When cases started to surge, there was no space available in public or private hospitals, leading to potentially avoidable loss of life. Initially, contact tracing was performed by the central government and later by the local governments; the formation of CICT teams made these operations more efficient given the limited manpower and complex logistics. Teams were formed locally with female community health volunteers, and social organizations were also engaged for contact tracing.

COVID-19 vaccination started on 27 January 2021, using the Oxford AstraZeneca vaccine, designated AZD1222 and sold under the brand name COVISHIELD. Although vaccine availability was a major concern when the Serum Institute India stopped supplying COVISHIELD in Nepal until 2022 [33,34], COVID-19 vaccinations continued, and substantial coverage was achieved among different age groups, including children aged 12–17 years old via the introduction of multiple vaccines. Despite this, Nepal has not begun vaccinating children below 12 years, which must begin as early as possible to prevent transmission among the youngest individuals [35]. Properly identifying key shortcomings and gaps and making necessary improvements in the health care delivery system are of the utmost importance to fight the possible next wave or a future, similar health crisis [22].

However, there were gaps at all stages of the response, including insufficient laboratory services, inefficient contact tracing, and poor logistics, coordination, and case management. Overall, Nepal was unprepared to respond to this unprecedented pandemic, and poor management led to potentially avoidable loss of life.

## 5. Conclusions

The GoN took immediate action, supported by the WHO and international partners, after the first domestic case of COVID-19 was reported. Laboratories were supported to become capable of testing for SARS-CoV-2 in Nepal a week after the first case was reported. The HLCC, led by the Deputy Prime Minister, and the CCMC were formed and made a series of important decisions to break the transmission chain and limit the spread of COVID-19 in Nepal. The MoHP acted immediately to fortify Nepal’s efforts to prevent the spread of the COVID-19 virus, such as strengthening the health desks at international and domestic airports and at border crossings between India, China, and Nepal.

To coordinate efforts, the ICS was activated by the MoHP and cluster meetings were initiated. Contract tracing was initially performed by the EDCD via teams that included medical doctors, public health experts, and laboratory staff. This task was later coordinated with the provincial and local governments as the number of cases surged. Efforts to share information with the public and communicate risks were initiated. Hospitals were designated for COVID-19 treatment and specific ambulance services were utilized to transfer patients. Quarantine and isolation centers were created by all three tiers of government; local governments were very proactive regarding quarantine.

The lessons learned from the SARS-CoV-2 pandemic will serve to inform planning for any future outbreak of novel high-impact respiratory pathogens. The national system of Nepal could have responded more efficiently and there is a need for a new framework that provides commitment to prompt detection and fully transparent, harmonized, coordinated, and timely communications and responses.

## Data Availability

Not applicable.
